# Supplementary figures and images for: A ‘Candidatus Liberibacter asiaticus’ effector SDE2470 facilitates citrus transcription factor CsVOZ2 degradation via BRUTUS E3 ligases
Source: PLoS Pathog. 2025 Dec 19;21(12):e1013797. doi: 10.1371/journal.ppat.1013797 (PMC12753073; doi:10.1371/journal.ppat.1013797)

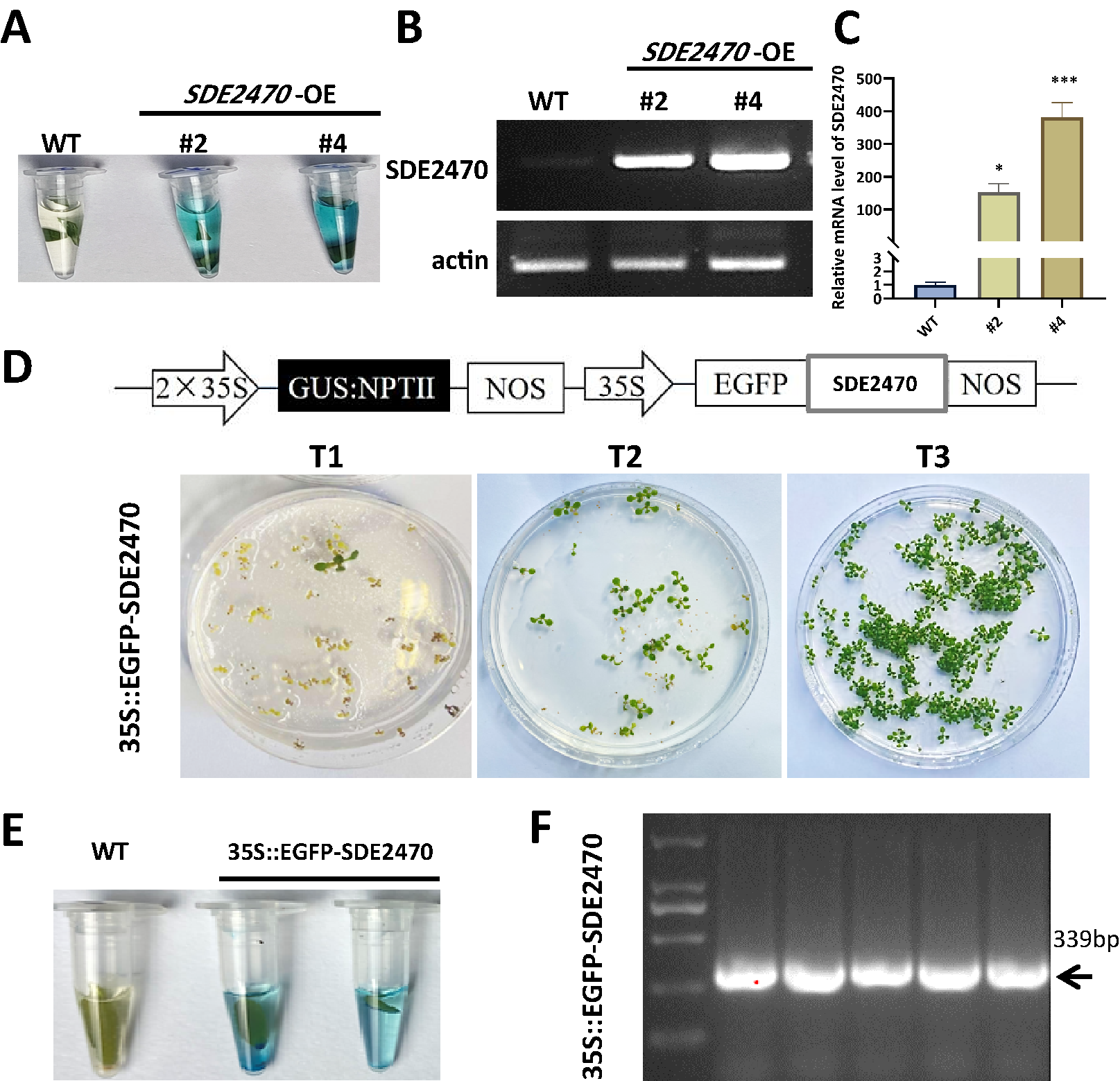

Supplement: S1 Fig — (A-C) GUS staining, PCR detection and RT-qPCR verification of SDE2470-OE transgenic citrus plants. The relative expression level of SDE2470 was moralized to citrus actin 7 and statistical significance was determined using one-way ANOVA test (*P < 0.05 and ****P < 0.0001). (D-F) Kanamycin resistance selection, GUS staining and PCR verification of T3 SDE2470-OE transgenic Arabidopsis thaliana plants. (TIF) [file ppat.1013797.s001.tif]

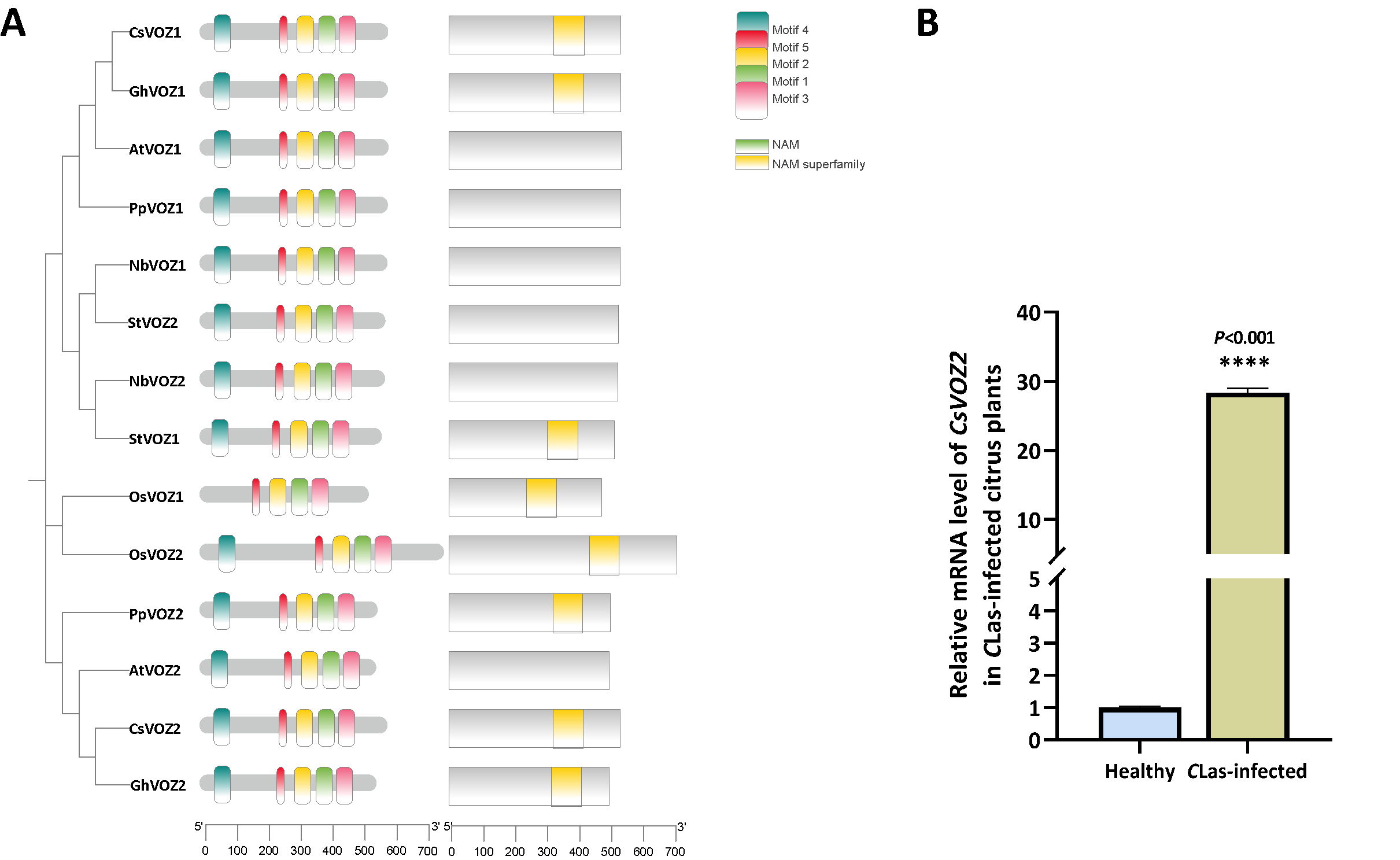

Supplement: S2 Fig — Phylogenetic analysis of CsVOZ2 and its homologs from other plants. Relative expression of CsVOZ2 in response to CLas infection in citrus plants. (TIF) [file ppat.1013797.s002.tif]

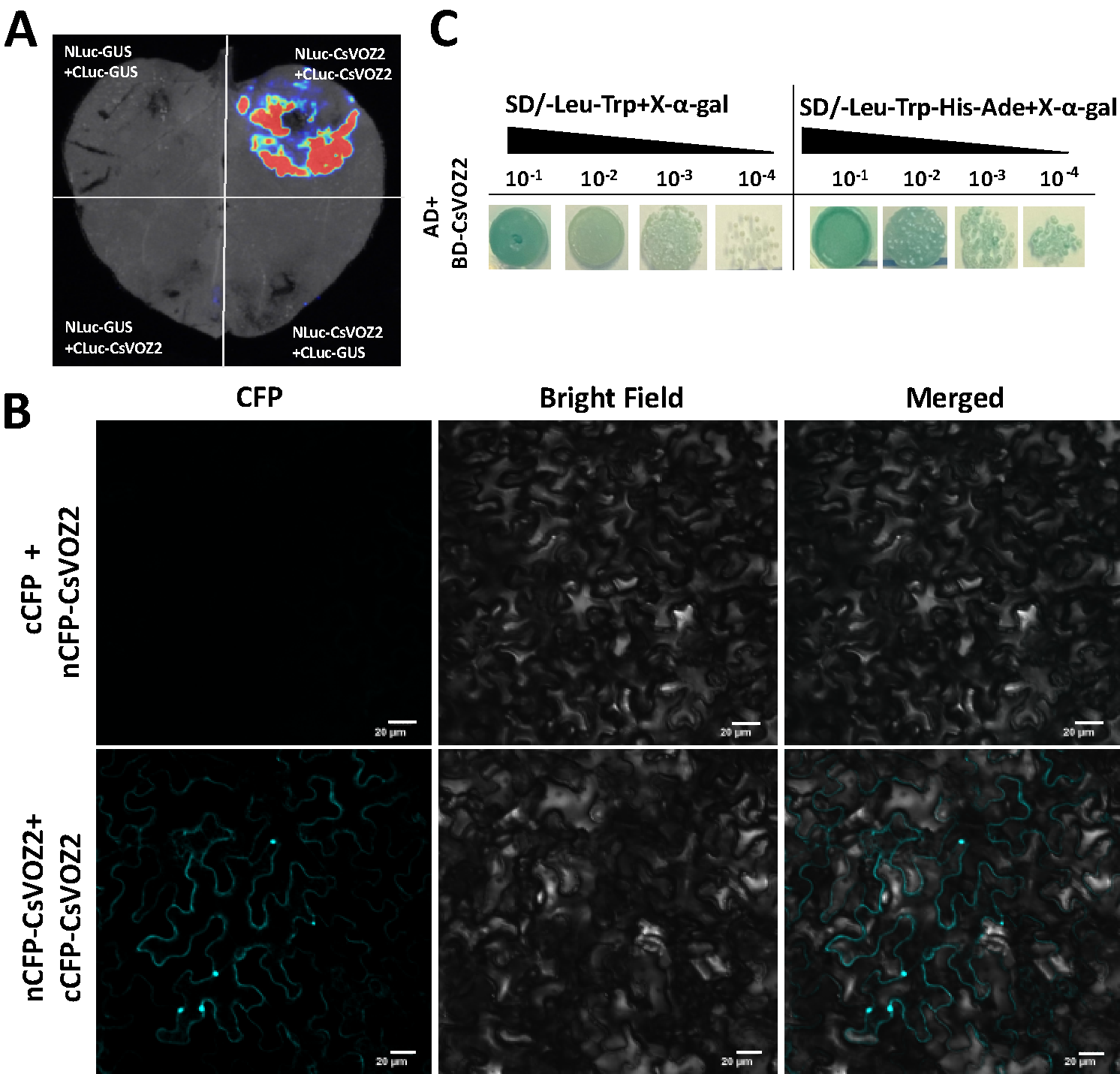

Supplement: S3 Fig — (A-B) The homodimer formation of CsVOZ2 was verified by LCA and BiFC. (C) Transcriptional activation activity assay of CsVOZ2 in yeast via Y2H system. BD-CsVOZ2 and AD empty vector were co-transformed into yeast strain and cultured in SD/-Leu-Trp + X-α-gal and SD/Leu-Trp-His-Ade + X-α-gal plates (lacking leucine, tryptophan, histidine and adenine and supplemented with X-α-gal). (TIF) [file ppat.1013797.s003.tif]

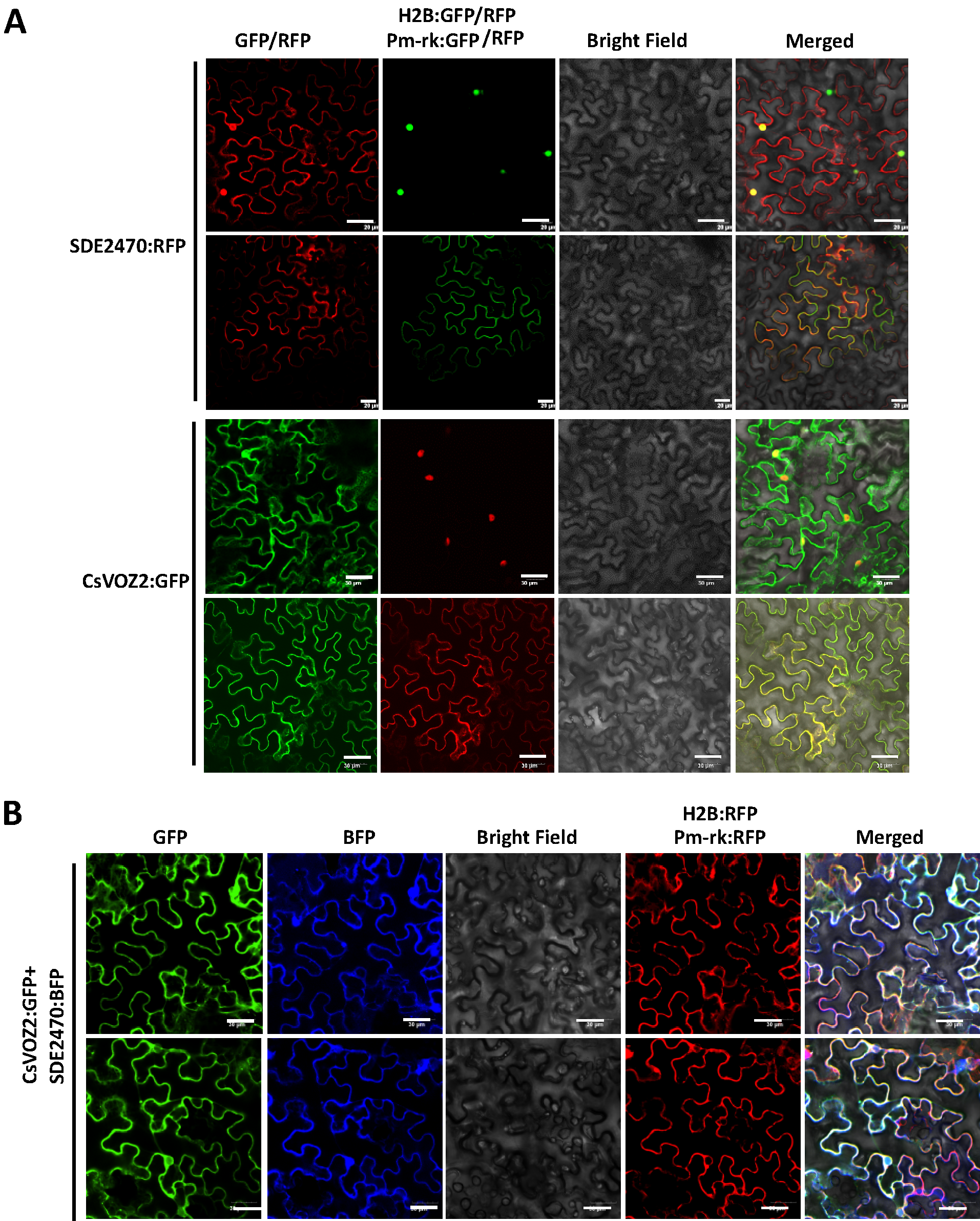

Supplement: S4 Fig — (A) Subcellular localization of SDE2470 and CsVOZ2 individually by transient co-expression with H2B-mCherry or cytoplasmic marker mCherry in N. benthamiana leaves. Scale bars = 20 μm. (B) Co-subcellular localization of SDE2470 and CsVOZ2 by transient co-expression with H2B-mCherry or cytoplasmic marker mCherry in N. benthamiana leaves. Scale bars = 20 μm. (TIF) [file ppat.1013797.s004.tif]

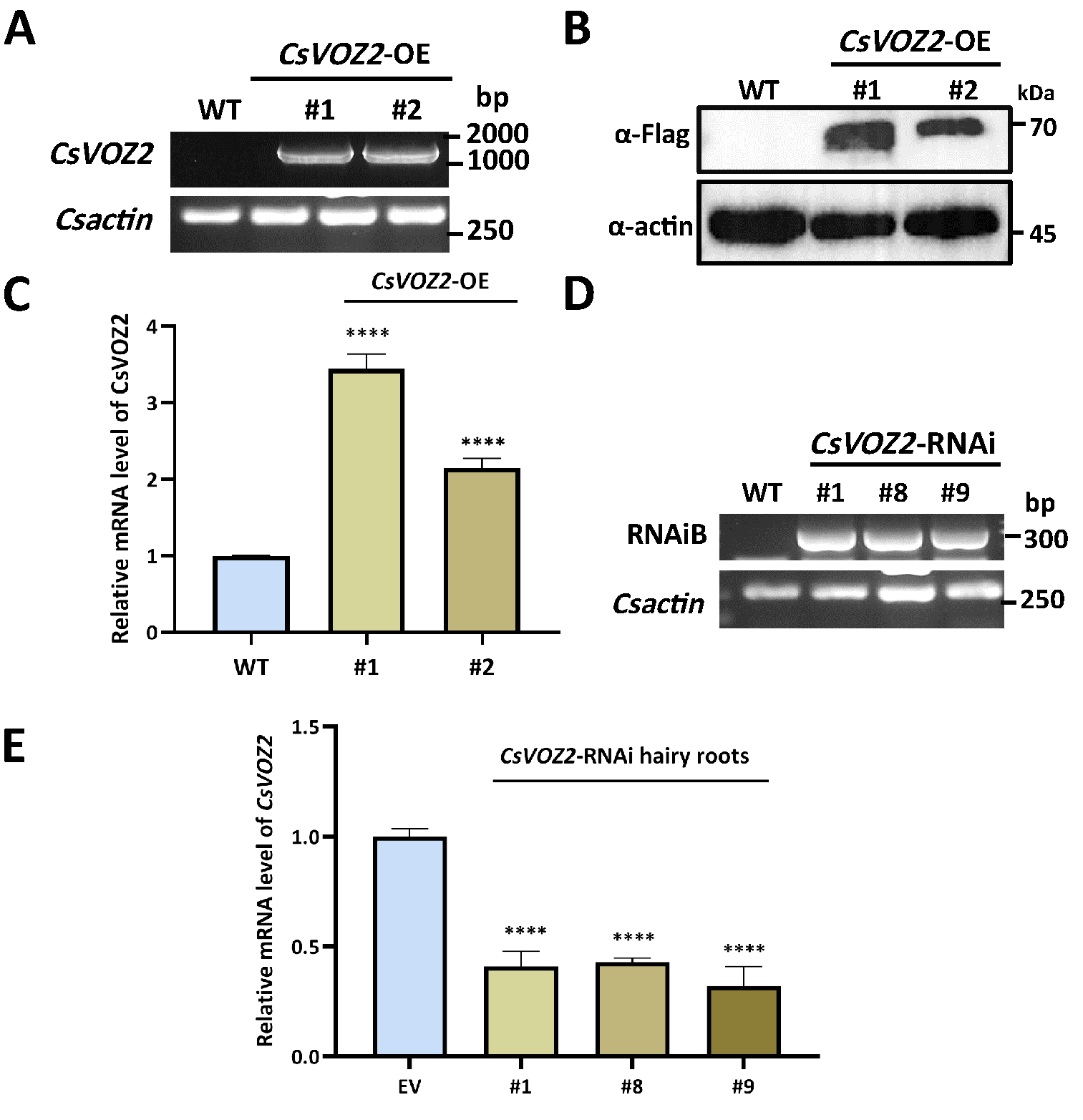

Supplement: S5 Fig — (A-C) Verification of CsVOZ2-OE transgenic citrus plants with PCR, WB and RT-qPCR assays. Actin served as a control. The relative expression level of CsVOZ2 was moralized to citrus actin and statistical significance was determined using one-way ANOVA test (****P < 0.0001). (D-E) Verification of CsVOZ2-RNAi citrus hairy roots with PCR and RT-qPCR assays. Actin served as control. The relative expression level of CsVOZ2 was moralized to citrus actin and statistical significance was determined using one-way ANOVA test (****P < 0.0001). (TIF) [file ppat.1013797.s005.tif]

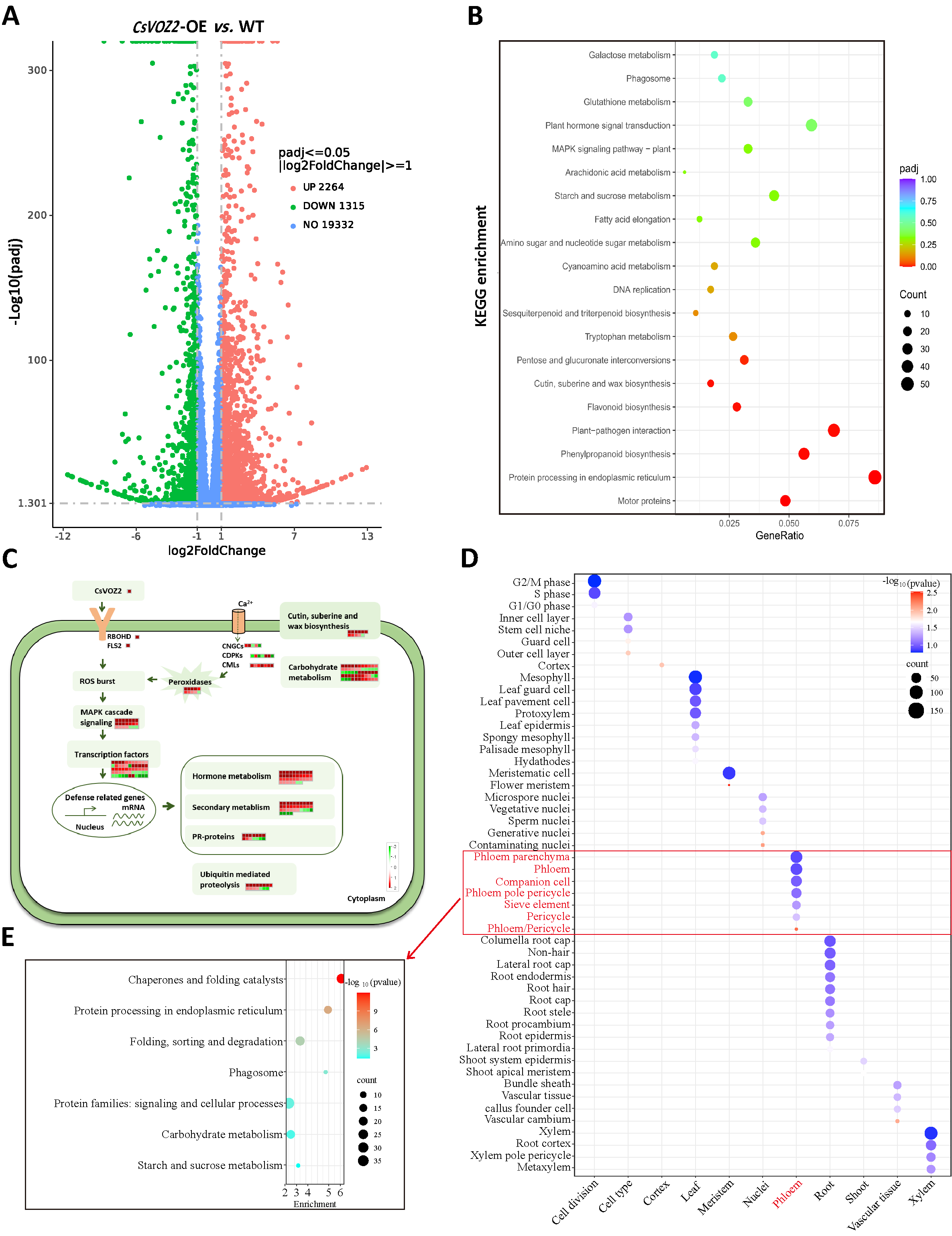

Supplement: S6 Fig — (A) Volcano plot displaying differentially expressed genes (DEGs) in healthy CsVOZ2-OE vs. WT plants. (B) Top 20 of DEGs enriched KEGG pathways in CsVOZ2-OE vs. WT plants. (C) Visualization of DEGs involved pathways in CsVOZ2-OE transgenic citrus plants. (D) Tissue-specific expression enrichment of DEGs in CsVOZ2-OE vs. WT plants. (E) Functional enrichment analysis of DEGs associated with the phloem complex. (TIF) [file ppat.1013797.s006.tif]

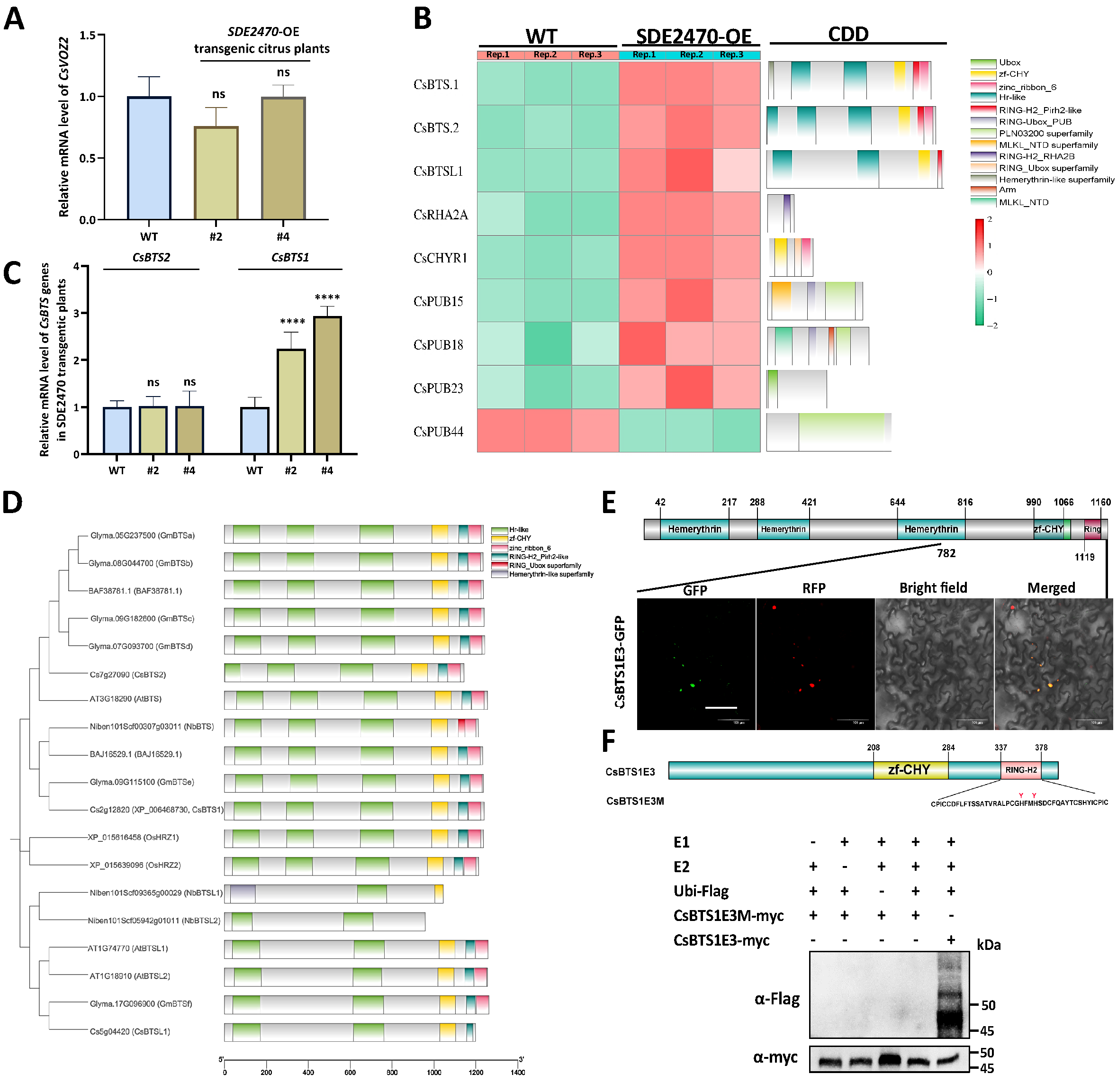

Supplement: S7 Fig — (A and C) Transcriptional levels of CsVOZ2 and CsBTS1/2 genes in SDE2470-OE transgenic citrus plants determined by RT-qPCR. The relative expression level of CsVOZ2 and CsBTSs was normalized to citrus actin and statistical significance was determined using one/two-way ANOVA (****P < 0.0001). (B) Heatmap of DEGs encoding E3 ligases in SDE2470-OE transgenic citrus plants via RNA-seq analyses. (D) Phylogenetic tree and domain architecture of CsBTSs homologues in plants. Multiple sequence alignment was performed using ClustalW. A phylogenetic tree was constructed with MEGA 11 based on 1,000 bootstrap replicates. Three CsBTS genes were retrieved from the Citrus sinensis V1 genome in the Citrus Pan-genome2breeding Database. For CsBTS1 (Cs2g12820), three protein isoforms were identified from the NCBI database and the one cloned (XP_006468730) in our study was used for phylogenetic construction. (E) Subcellular localization of CsBTS1E3. CsBTS1E3 (782–1242 aa) is a truncate of the full length CsBTS1 (XP_006468730) as indicated by the schematic diagram. (F) In vitro assay of E3 ligases activity of CsBTS1E3. Key residues critical for ligase activity were mutated (indicated in red in the schematic) to assess their functional role. (TIF) [file ppat.1013797.s007.tif]

**Fig 2C**

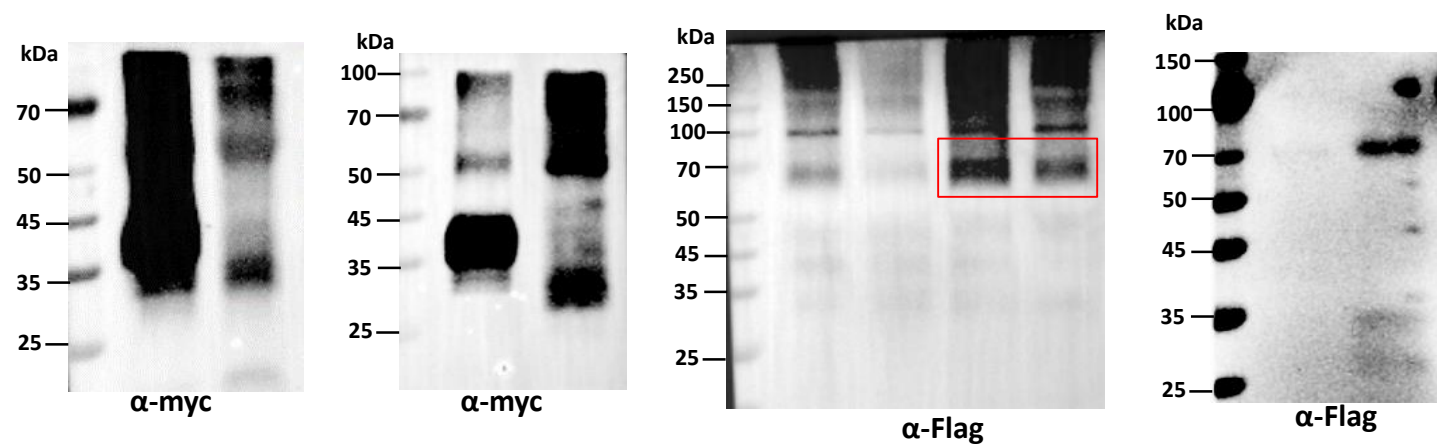

**Fig 2D**

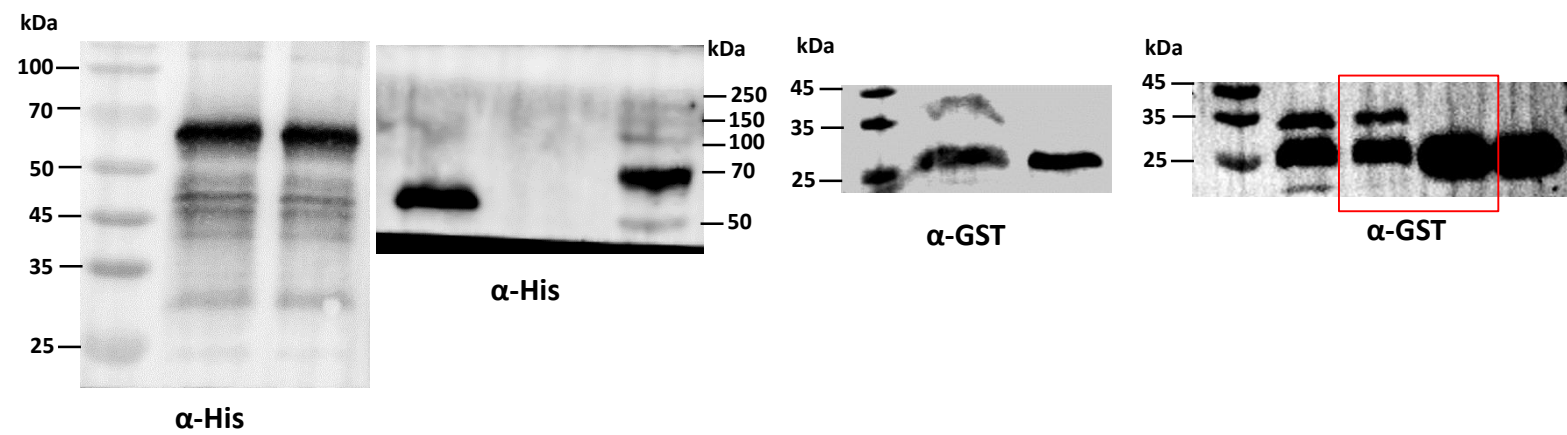

**Fig 4A**

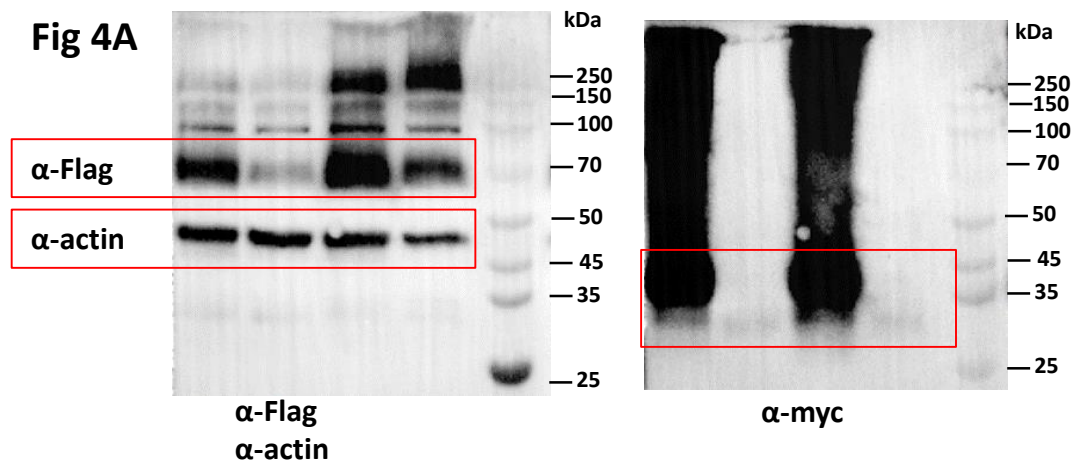

**Fig 4D**

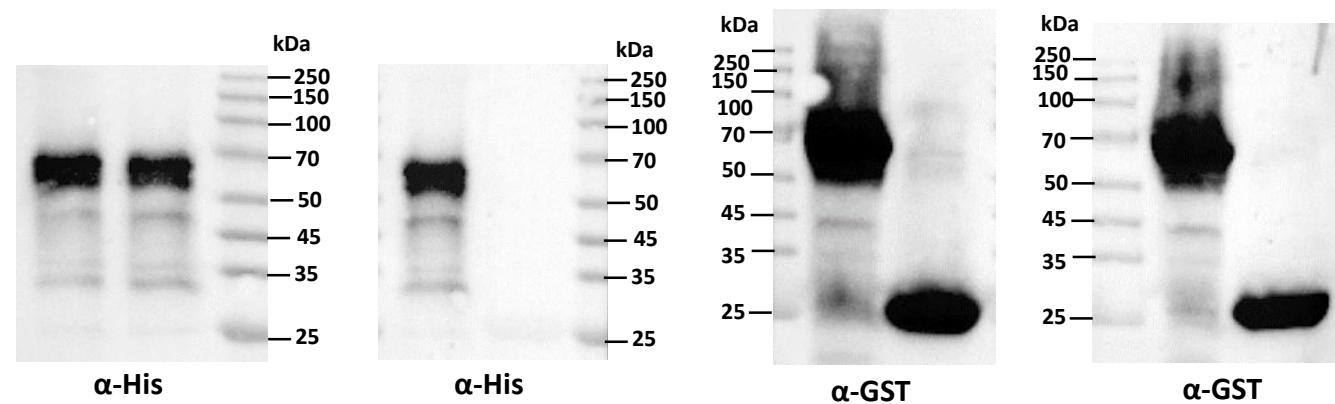

**Fig 4E**

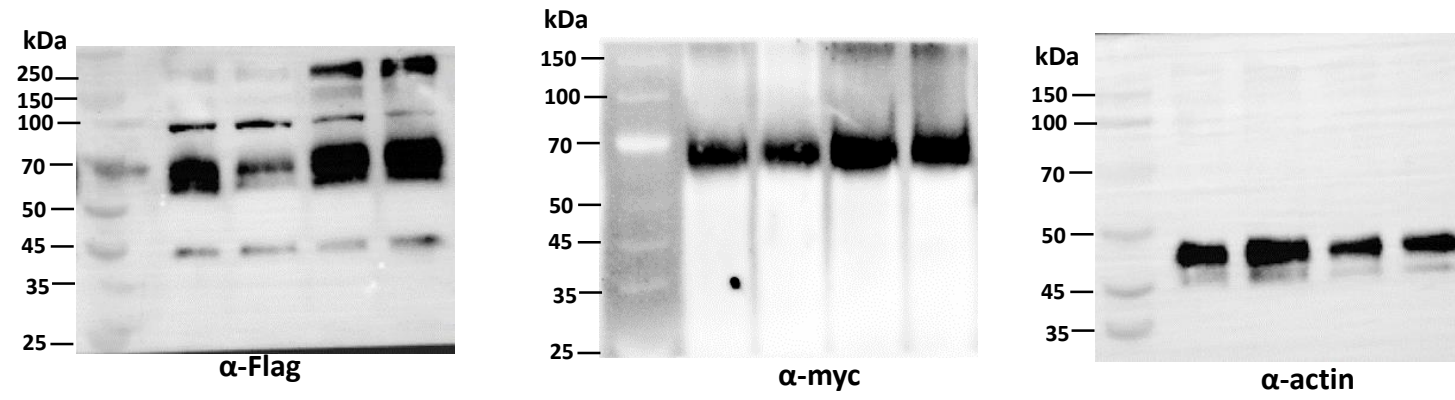

**Fig 4F**

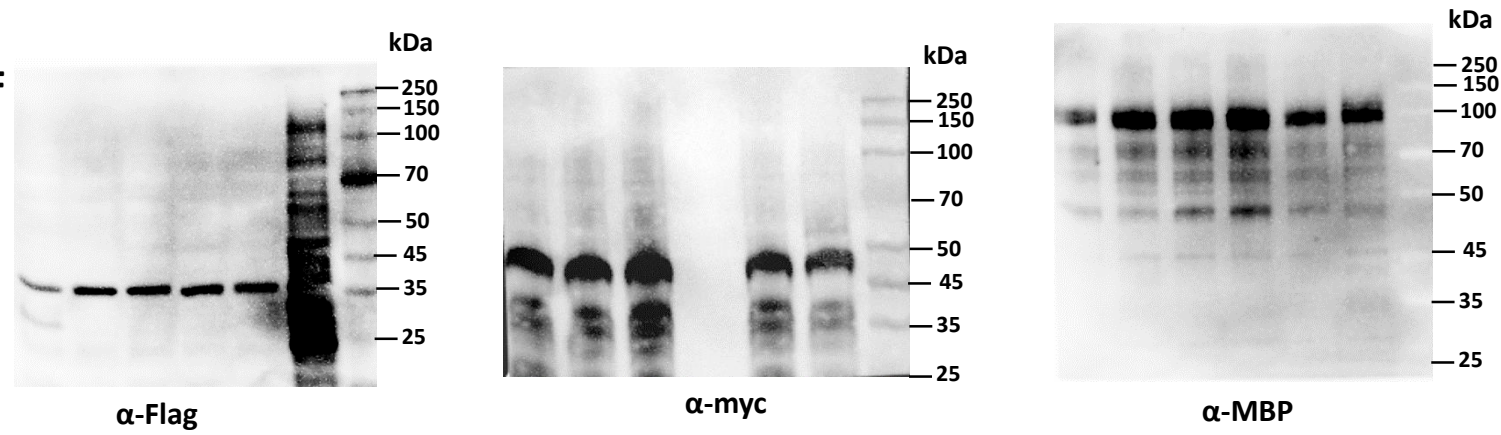

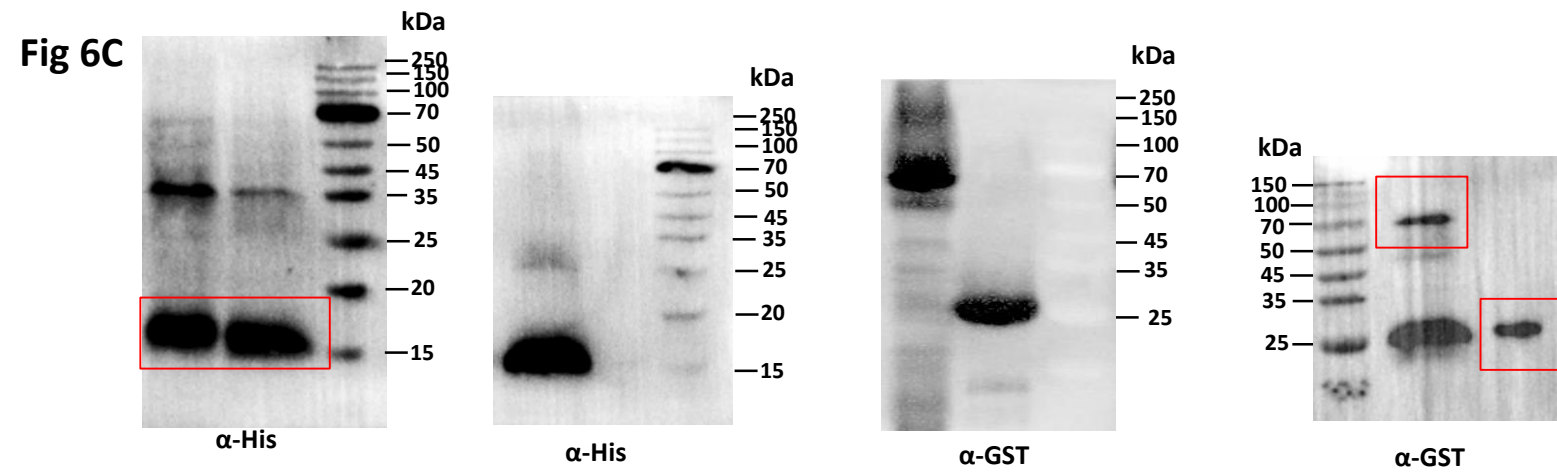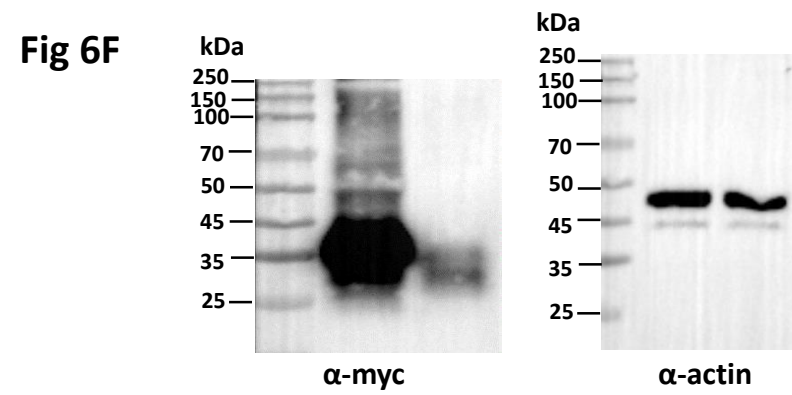

**Fig 7A**

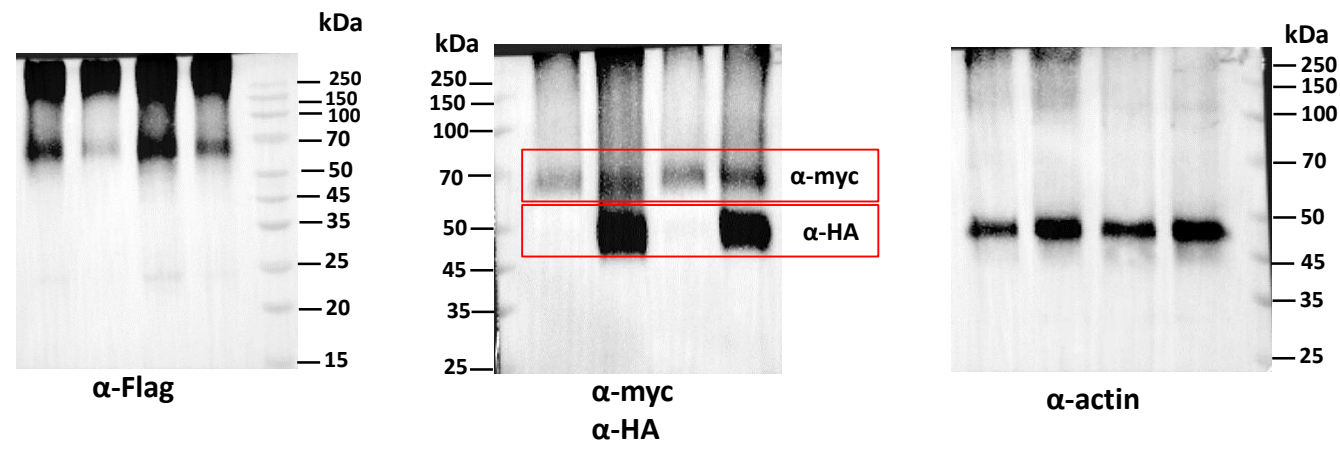

**Fig 7B**

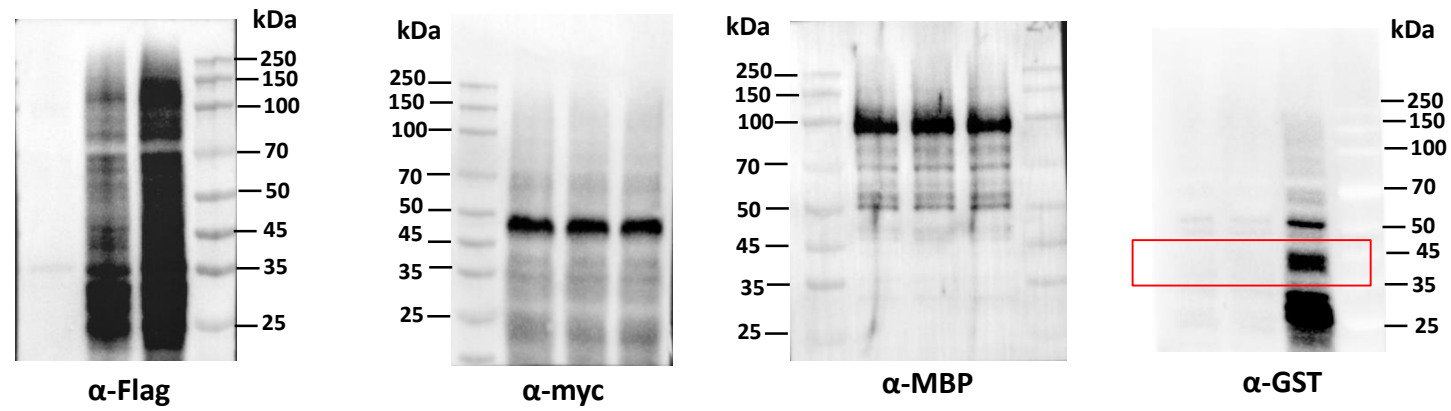

**Fig S5B**

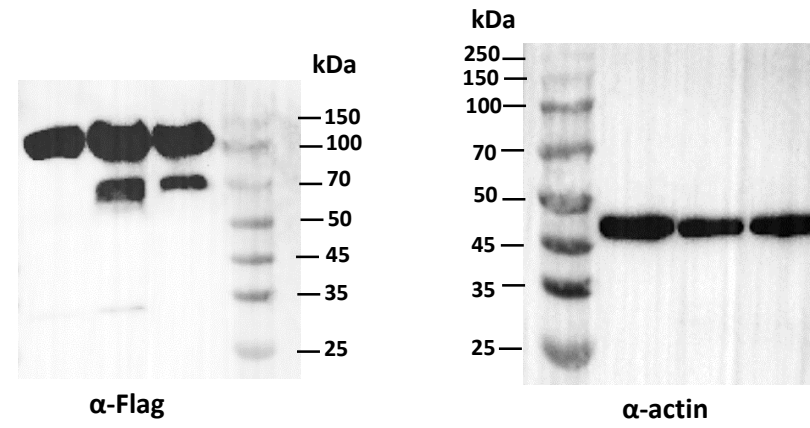

**Fig S7F**

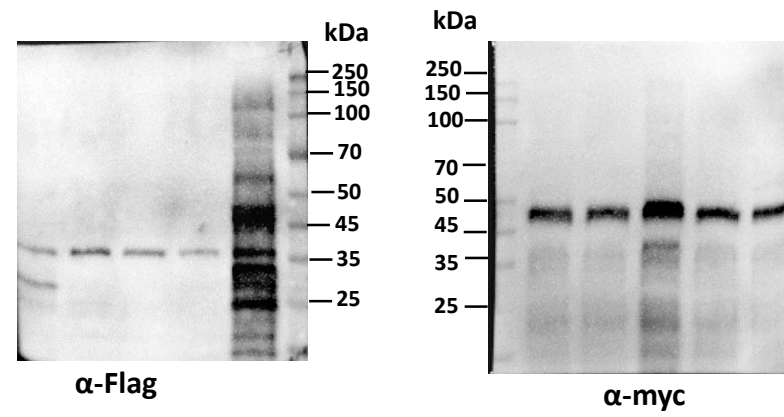

Supplement: S1 Data — (PDF) [file ppat.1013797.s012.pdf]
