# Supplementary material for: A ‘Candidatus Liberibacter asiaticus’ effector SDE2470 facilitates citrus transcription factor CsVOZ2 degradation via BRUTUS E3 ligases
Source: PLoS Pathog. 2025 Dec 19;21(12):e1013797. doi: 10.1371/journal.ppat.1013797 (PMC12753073; doi:10.1371/journal.ppat.1013797)
Supplement: Attachment — Submitted filename: Comments to Author.docx [file ppat.1013797.s013.docx]

**Reviewer’s Comments**

The authors investigated the role of the *Candidatus Liberibacter asiaticus* (CLas) effector SDE2470 in citrus Huanglongbing (HLB) pathogen. They demonstrated that SDE2470 physically interacts with the citrus transcription factor CsVOZ2 and promotes its proteasomal degradation via the E3 ligase CsBTS1. Overexpression of CsVOZ2 enhanced citrus resistance to CLas, while its knockdown increased susceptibility. Mechanistically, SDE2470 strengthened the CsBTS1–CsVOZ2 interaction and boosted CsBTS1’s ubiquitin ligase activity, thereby suppressing ROS- and ABA-mediated defense pathways and facilitating bacterial infection. However, the manuscript requires substantial improvement in language clarity (many grammatical errors and redundancies).

**Major Comments**

1. Many results mention qPCR, WB, RNA-seq etc., but you don’t specify the number of biological replicates in the Results section (only sometimes in Methods). These need consistency.
2. Figures are said to be supported by statistical analysis (e.g., Fig 3D, Fig 5D), but the text doesn’t always describe statistical tests or significance levels.
3. In Discussion (p.12–14), you link CsVOZ2 to phloem carbohydrate metabolism, but the evidence is indirect (based on RNA-seq prediction). Phrase more cautiously.
4. The introduction lists many effectors in a very long paragraph. Breaking into subparagraphs (effector categories, classical vs non-classical, functions) would improve readability.
5. Needs rephrasing “Therefore, SDE2470 served as a board basal immune suppressor to facilitate pathogen infection”
6. In Results, you mention failure to clone full-length CsBTS1 but used truncation (E3 domain). Discuss limitations this causes in interpretation.
7. Some results mention supplementary figures (S7B, S7D etc.) but no description in text beyond “data shown.” Expand slightly.
8. Simplify language further for non-specialist readers. Phrases like “hijacks a plant protein” are fine, but “CsVOZ2 made plants more resistant” could be rephrased more clearly.

**Minor Comments**

1. “These studies have elucidated the molecular mechanisms. SDE1…” to combine into one sentence or rephrase for flow.
2. Typo: “share 55~61% amino acid sequence similarity” to “shares 55–61%” (and use en dash).
3. Acronyms: define ROS, ABA, UPS at first mention in Introduction, not later.
4. “were tested on SDE2470-overexpressing (OE) transgenic Arabidopsis and citrus plants” to rephrase as “were tested in SDE2470-overexpressing…”
5. Sentence: “Although CLas was detected in both WT and CsVOZ2-OE plants…” better as “CLas was detected in both, but its population was significantly lower in CsVOZ2-OE plants.”
6. Inconsistency: “26 proteasome pathway” vs. “26S proteasome pathway.” Use “26S” consistently.
7. Replace “in negative regulation of host immunity” to “negatively regulates host immunity.”

**Please cite these references**

- Ghosh, D. K., Bhose, S., Warghane, A., Motghare, M., Sharma, A. K., Dhar, A. K., & Gowda, S. (2016). Loop-mediated isothermal amplification (LAMP) based method for rapid and sensitive detection of ‘Candidatus Liberibacter asiaticus’ in citrus and the psyllid vector, Diaphorina citri Kuwayama. *Journal of Plant Biochemistry and Biotechnology*, *25*, 219-223.
- Ghosh, D. K., Kokane, S. B., Kokane, A. D., Warghane, A. J., Motghare, M. R., Bhose, S., ... & Reddy, M. K. (2018). Development of a recombinase polymerase based isothermal amplification combined with lateral flow assay (HLB-RPA-LFA) for rapid detection of" Candidatus Liberibacter asiaticus". *PloS one*, *13*(12), e0208530.
- Ghosh, D., Kokane, S., Savita, B. K., Kumar, P., Sharma, A. K., Ozcan, A., ... & Santra, S. (2022). Huanglongbing pandemic: current challenges and emerging management strategies. *Plants*, *12*(1), 160.

**Major Revision Needed** The experiments are solid and novel, but the manuscript needs significant polishing in language, structure, and data reporting before it is suitable for publication in PLOS Pathogens.
