# Supplementary material for: A ‘Candidatus Liberibacter asiaticus’ effector SDE2470 facilitates citrus transcription factor CsVOZ2 degradation via BRUTUS E3 ligases
Source: PLoS Pathog. 2025 Dec 19;21(12):e1013797. doi: 10.1371/journal.ppat.1013797 (PMC12753073; doi:10.1371/journal.ppat.1013797)
Supplement: Attachment — Submitted filename: Response to Reviewers.docx [file ppat.1013797.s014.docx]

**Dear Reviewers,**

We sincerely thank you for your valuable and great comments, which have helped us significantly improve our manuscript. We have revised the manuscript thoroughly to address all the points raised and have provided a point-by-point response below.

**Part I – Summary**

**Reviewer #1**: The Candidatus Liberibacter asiaticus (CLas) caused Citrus Huanglongbing (HLB) is a destructive disease in citrus industry worldwide. This manuscript provides novel and valuable insights into the role of a CLas effector (SDE2470) and its interactions with interaction with host CsVOZ2 and CsBTS1, providing mechanistic insight into host–pathogen interplay. The integration of yeast two-hybrid, BiFC, co-IP, RNAi, and VIGS approaches demonstrates a strong experimental framework. Findings of this study are significant to the citrus HLB field, as they highlight how effectors manipulate host protein networks beyond defense suppression, possibly rewiring transcriptional regulation and signaling. Although the link between CsVOZ2/CsBTS1 regulation and HLB disease progression remains correlative, mechanistic depth could be improved by showing downstream signaling/transcriptome effects.

**Response**: We appreciate this helpful comment. Accordingly, we have revised the paragraph concerning transcriptional regulation and signaling to include a detailed analysis of the *CsVOZ2*-OE transcriptome profile and its downstream regulatory functions (**lines 175-199**). To complement this analysis, we have provided a diagram illustrating the regulated pathways of the DEGs (revised **S6C Fig**).

**Reviewer #2**: The authors investigated the role of the Candidatus Liberibacter asiaticus (CLas) effector SDE2470 in citrus Huanglongbing (HLB) pathogen. They demonstrated that SDE2470 physically interacts with the citrus transcription factor CsVOZ2 and promotes its proteasomal degradation via the E3 ligase CsBTS1. Overexpression of CsVOZ2 enhanced citrus resistance to CLas, while its knockdown increased susceptibility. Mechanistically, SDE2470 strengthened the CsBTS1–CsVOZ2 interaction and boosted CsBTS1’s ubiquitin ligase activity, thereby suppressing ROS- and ABA-mediated defense pathways and facilitating bacterial infection. However, the manuscript requires substantial improvement in language clarity (many grammatical errors and redundancies).

**Response**: Suggestion was taken. We have conducted a thorough revision of the manuscript and made extensive corrections to address language issues. Please check the main text.

**Reviewer #3**: Based on previous studies identifying SDE2470 as a virulence factor of CLas, Fu et al. identified CsVOZ2 as an interactor of SDE2470 and demonstrated its role in suppressing CLas infection. Their findings revealed that CsVOZ2 was associated with plant defense mechanisms. Further investigation showed that CsVOZ2 interacted with CsBTS1, an E3 ubiquitin ligase that mediated the degradation of CsVOZ2 via the 26S proteasome pathway. The authors proposed that SDE2470 enhanced the E3 ligase activity of CsBTS1 through their interaction, thereby strengthening the CsBTS1-CsVOZ2 association and promoting CsVOZ2 degradation. This process ultimately compromised plant defense and facilitates CLas infection.

**Part II – Major Issues: Key Experiments Required for Acceptance**

**Reviewer #1: Major suggestion**

If possible, examined expression of well-characterized defense-related genes (e.g., PR1, NPR1-related pathways, JA/SA markers) in CsVOZ2/CsBTS1-silenced plants with or without effector expression, to directly link interactions with immune outcomes.

**Response**: Suggestion was taken. The well-characterized marker genes were assayed and they were consistently upregulated in healthy *CsVOZ2*-OE transgenic citrus plants, but downregulated in healthy RNAi citrus hairy roots (revised **Fig 3D**). Constitutive expression of either *CsBTS1E3* or its mutant form (*CsBTS1E3M*) in citrus hairy roots also led to a general reduction in the expression of these markers (revised **Fig 5E**). This observation highly supports their biological roles during *C*Las infection.

**Reviewer #2: Major Comments**

1. Many results mention qPCR, WB, RNA-seq etc., but you don’t specify the number of biological replicates in the Results section (only sometimes in Methods). These needs consistency.

**Response**: Sorry for the inconsistency. For *C*Las susceptibility assays and qPCR, at least two independent transgenic lines were used for both citrus plants (with three to four replicates for each line) and hairy roots. RNA-seq was performed using the *CsVOZ2*-OE #2 line with three biological replicates. WB experiments were repeated at least three times. For all other experiments, the number of biological or technical replicates is indicated in the corresponding figure legends, and further details can be found in the main text.

2. Figures are said to be supported by statistical analysis (e.g., Fig 3D, Fig 5D), but the text doesn’t always describe statistical tests or significance levels.

**Response**: OK. A new paragraph describing the statistical analyses was added to the end of the Materials and Methods section, and the respective statistical tests and significance level are now specified in corresponding figure legends.

3. In Discussion (p.12–14), you link CsVOZ2 to phloem carbohydrate metabolism, but the evidence is indirect (based on RNA-seq prediction). Phrase more cautiously.

**Response**: We appreciate the reviewer's valuable suggestion. While our RNA-seq profiling indicated that *CsVOZ2*-OE activated multi-layered defense responses, including carbohydrate metabolism, we agree that this specific point lacked supporting wet-lab experimental evidence. Therefore, the corresponding statement has been revised in the Discussion section.

4. The introduction lists many effectors in a very long paragraph. Breaking into subparagraphs (effector categories, classical vs non-classical, functions) would improve readability.

**Response**: We have condensed and merged the paragraphs to eliminate redundancy.

5. Needs rephrasing “Therefore, SDE2470 served as a board basal immune suppressor to facilitate pathogen infection”.

**Response**: The sentence was rephrased as “Therefore, SDE2470 served as a board-spectrum suppressor of basal immunity to facilitate pathogen infection”.

6. In Results, you mention failure to clone full-length CsBTS1 but used truncation (E3 domain). Discuss limitations this causes in interpretation.

**Response**: Thank you for the helpful suggestion. In response, a phylogenetic tree of CsBTSs retrieved from citrus database were constructed (revised **S7D Fig**). To facilitate interpretation, a schematic of the CsBTS1E3M construct that highlights the mutated sites, presented alongside its ligase activity assay (**revised S7F Fig**). As recommended, the potential difficulties in cloning full-length CsBTS1 was also discussed (**lines 336-344**).

7. Some results mention supplementary figures (S7B, S7D etc.) but no description in text beyond “data shown.” Expand slightly.

**Response**: Thanks for this helpful comment. We have now expanded the descriptions of the supplementary figures throughout the main text.

8. Simplify language further for non-specialist readers. Phrases like “hijacks a plant protein” are fine, but “CsVOZ2 made plants more resistant” could be rephrased more clearly.

**Response**: Alright. The ‘Author summary’ section was revised and simplified.

**Reviewer #3: Major comments**

While this story is interesting, the underlying logic and experimental validation require further reinforcement to be fully convincing.

**1.** SDE2470, CsVOZ2 and CsBTS1 interacted with each other, why did not CsBTS1 mediate SDE2470 degradation, but CsVOZ2?

**Response**: E3 ligase-substrate interaction does not always guarantee degradation. For example, the rice BTB-type E3 ligase OsNPR1-3E3 binds OsNRR and OsrTGA2.1 without promoting their degradation (Wang et al., 2022). Consistently, our E3 ligase screen revealed several interactors of CsVOZ2 that did not trigger its turnover. These observations reinforce the concept that E3 ligases can function beyond proteolysis, such as in complex assembly or allosteric regulation. In the case of CsBTS1 and SDE2470, their interaction may likewise serve a non-degradative role-for instance, as a scaffold in a larger protein complex. The absence of degradation could stem from a lack of ubiquitin sites on SDE2470, or because the interaction enables other regulatory modifications. Nerveless, I am not sure what the detailed mechanism at this point.

**Reference**

Wang R, You X, Zhang C, Fang H, Wang M, Zhang F, et al. An ORFeome of rice E3 ubiquitin ligases for global analysis of the ubiquitination interactome. Genome Biol. 2022;23(1):154. doi: 10.1186/s13059-022-02717-8.

**2.** Several assays were performed in the artificial materials, like N. benthamiana, and overexpression plants, but not in the CLas-infected plants. For example, the expression of genes, the ubiquitination test, protein-protein interaction. Lines 192-195, when RT-qPCR assay showed no change in CsVOZ2 gene expression on SDE2470-OE plants, it is illogical to test the CsVOZ2 protein level in N. benthamiana.

**Response**: Really appreciate your great questions. To delineate the specific role of the SDE2470 effector, the expression of both *CsVOZ2* and *CsBTS1* genes were assessed in stable *SDE2470*-OE citrus plants rather than the complex *C*Las infection context. Indeed, to directly address your point, we have also measured their expression in *C*Las-infected WT plants via RT-qPCR and found their upregulation, with a particularly pronounced induction of *CsVOZ2*. In contrast, the expression of *CsBTS1* was only slightly induced, but this change was not statistically significant at the level 0.05 (**Figure 1 in response file**).

Regarding the use of *N. benthamiana*, this is a well-established heterologous system for high-efficiency transient protein expression, which we employed specifically for our protein-protein interaction and degradation assays.

The absence of significant *CsVOZ2* mRNA changes in *SDE2470*-OE citrus plants is likely attributable to its rapid turnover in the absence of *C*Las challenge. However, the lack of mRNA-level regulation does not preclude effects at the protein level. It is well-established that pathogen effector often modulate host targets through post-translational mechanisms, including altering protein stability or subcellular localization.

**Figure 1. The expression of *CsVOZ2* and *CsBTS1* genes in response to *C*Las infection**

**3.** A key aspect of this story was the transcriptional activation capability of CsVOZ2; however, the evidence provided remains limited. Fig. S3A and S3B only demonstrate self-interaction of CsVOZ2, without confirming the formation of homodimers. Additionally, Fig. S3C illustrates self-interaction in a yeast two-hybrid assay but does not substantiate transcriptional activation activity in a yeast one-hybrid context. Therefore, the claim that “CsVOZ2 is a truly transcription factor as homodimers” (lines 148-150) appears overstated. Further evidence is necessary to conclusively establish the transcriptional activation function of CsVOZ2.

**Response**: Thanks for your comments. As shown in Fig. S3A-S3B, CsVOZ2 exhibited self-interaction, suggesting its ability to form homodimers. In Fig. S3C, co-transformation of BD-CsVOZ2 with the AD empty vector enabled yeast growth in the Y2H system, indicating that CsVOZ2 possesses the transcriptional activation activity, a prerequisite in activating gene expression. The Y1H assay was not performed, as CsVOZ2 was not involved in interacting with any specific gene promoter in current study. To be more accurate, we have revised the statement to: “CsVOZ2 is capable of forming homodimers and exhibits transcriptional activation activity.”

**4.** There are some flaws in Fig. 7B. What did MBP antibody detect for? Was some protein missing? Why could myc antibody detect a band without myc-CsBTS1E3 in the first lane?

**Response**: We apologize for the confusion in the original Fig. 7B. The MBP antibody was used to detect the substrate MBP-tagged CsVOZ2. The first lane did contain Myc-CsBTS1E3 as intended, which is essential for comparison with the second lane. This was a labeling error and has now been corrected. To ensure the robustness of the data, the experiment was repeated, and a revised **Fig. 7B** is now presented.

**Part III – Minor Issues: Editorial and Data Presentation Modifications**

**Reviewer #1: Editorial and minor suggestion**

1. Line 15 "remains difficult to manage" to "lacks effective control strategies"

**Response**: Suggestion was taken.

2. Line 57-75 Streamline the background on CLas effectors to avoid redundancy and transition more directly to the rationale for studying SDE2470. In particular, the function of SED5 (same as SDE2470 in this study) have been previously reported in Zhao et al 2025 (doi: 10.1126/science.adq7203). It is suggested to add more detail of previous work related to SDE5 or SDE2470 in this section.

**Response**: The background section on *C*Las effectors has been streamlined and condensed. Additionally, more information regarding SDE5 (SDE2470) has been included with a new one recently published (Zhao *et al*., 2025a).

Zhao P, Sun Y, Chen X, et al., 2025a. A small peptide APP3-14 disrupts pathogen-insect mutualism via modulating plant MYC2-mediated olfactory defense. Plant Commun, 101544.

3. Line 149 "harbors transcriptional activation activity" to "exhibits transcriptional activation activity".

**Response**: OK, corrected.

4. Line 165 "...was significantly lower in CsVOZ2-OE than that in WT plants..." to "...was significantly lower in CsVOZ2-OE plants than in WT plants..."

**Response**: OK, correction was accecpted.

5. Line 166 "whereas CLas colonizes much faster..." to "CLas colonized more rapidly"

**Response**: Revised.

6. Line 243 "...enhances CsBTS1E3 degradation on CsVOZ2..." to "enhances CsBTS1E3-mediated degradation of CsVOZ2"

**Response**: OK. Thanks for the valuable correction.

7. Line 311 "deciphering how this bacterium manipulates..." to "deciphering how CLas manipulates...

**Response**: Changed.

8. The legends of some figures can be improved. Such as explicitly state whether data are from citrus or N. benthamiana, number of replicates, type of statistical test used.

**Response**: Thanks for the suggestion. The figure legends have been revised thoroughly, specified plant source, number of replicates and type of statistical test.

**Reviewer #2: Minor Comments**

1. “These studies have elucidated the molecular mechanisms. SDE1…” to combine into one sentence or rephrase for flow.

**Response**: This paragraph has been revised and concise in the Introduction section.

2. Typo: “share 55~61% amino acid sequence similarity” to “shares 55–61%” (and use en dash).

**Response**: Ok, revised.

3. Acronyms: define ROS, ABA, UPS at first mention in Introduction, not later.

**Response**: OK. ROS, ABA and UPS were all defined in the Introduction.

4. “were tested on SDE2470-overexpressing (OE) transgenic Arabidopsis and citrus plants” to rephrase as “were tested in SDE2470-overexpressing…”

**Response**: Ok, revised.

5. Sentence: “Although CLas was detected in both WT and CsVOZ2-OE plants…” better as “CLas was detected in both, but its population was significantly lower in CsVOZ2-OE plants.”

**Response**: Thanks, suggestion accepted.

6. Inconsistency: “26 proteasome pathway” vs. “26S proteasome pathway.” Use “26S” consistently.

**Response**: Ok, ‘26S’ was consistently used.

7. Replace “in negative regulation of host immunity” to “negatively regulates host immunity.”

**Response**: Ok, revised.

**Reviewer #3: Minor comments:**

1. Why were the positions of 358 and 361 in CsBTS1E3M mutated?

**Response**: It’s a good question. E3 ligases are classified into distinct subfamilies based on their conserved domains, such as RING, HECT, RBR, and U-box. CsBTS1E3 contains a conserved RING_H2 motif (**S7E Fig**), which has been identified previously with a catalytic site centered on a histidine (H) residue (Shen et al., 2016). Therefore, we mutated both H residues to tyrosine (Y) in CsBTS1E3M construct (**S7F Fig**).

Shen Q, Hu T, Bao M, Cao L, Zhang H, Song F, et al. Tobacco RING E3 Ligase NtRFP1 Mediates Ubiquitination and Proteasomal Degradation of a Geminivirus-Encoded βC1. Mol Plant. 2016;9(6):911-25. doi: 10.1016/j.molp.2016.03.008.

2. The loading of reference proteins was different in this manuscript, such as Fig. 4C, 4B, and 7A. Fig. 7B.

**Response**: Fig. 7B is an *in vitro* assay and has been revised.

Figs. 4B, 4C, and 7A are *in vivo* assays. They demonstrate degradation effects in which we aimed to consolidate four treatments into a single figure for direct comparison. We have attempted diffident infiltration strategies. After finding that infiltrating different treatments into separate leaves or leaf halves yielded inconsistent protein expression, we implemented the third inoculation stagey: each leaf was divided into four sections (**Figure 2 in response file**), with each section receiving a different treatment. Protein samples were prepared by pooling equal weights (0.1 g) of tissue from the same section across multiple leaves, and equal volumes of extract were loaded for blotting without actin-based normalization.

Although minor loading variations are visible in the actin controls, the protein degradation trends do not affect the core conclusions. To more exactly address this point, the Flag-CsVOZ2 abundance was normalized to the corresponding actin abundance using ImageJ (detailed information was supplemented as **S3 Table**). When the ratio in the first lane was set to 1.00 for comparisons, the resulting data revealed a more statistically significant degradation pattern (Please refer to revised **Fig 4A, 4E and Fig 7A**). Consequently, we consider the current results to be conclusive and have not repeated the experiments at this stage. That said, we would be happy to perform additional repeats with adjusted loading if you deem it necessary.

**Figure 2. Schematic of the infiltration for protein degradation assays in *N. benthamiana*.**
